# Community-based exercise programs incorporating healthcare-community partnerships to improve function post-stroke: feasibility of a 2-group randomized controlled trial

**DOI:** 10.1186/s40814-022-01037-9

**Published:** 2022-04-22

**Authors:** Gayatri Aravind, Kainat Bashir, Jill I. Cameron, Jo-Anne Howe, Susan B. Jaglal, Mark T. Bayley, Robert W. Teasell, Rahim Moineddin, Joanne Zee, Walter P. Wodchis, Alda Tee, Susan Hunter, Nancy M. Salbach

**Affiliations:** 1 Department of Physical Therapy, University of Toronto, 160-500 University Avenue, Toronto, ON M5G 1V7 Canada; 2 Department of Occupational Science and Occupational Therapy, University of Toronto, 160-500 University Avenue, Toronto, ON M5G 1V7 Canada; 3 Toronto Rehabilitation Institute, University Health Network, University of Toronto, 550 University Avenue, Toronto, ON M5G 2A2 Canada; 4 The KITE Research Institute, University Health Network, 550 University Avenue, Toronto, ON M5G 2A2 Canada; 5 Department of Medicine, University of Toronto, C. David Naylor Building, 6 Queen’s Park Crescent West, Third Floor, London, ON M5S 3H2 Canada; 6 Schulich School of Medicine and Dentistry, Western University, St Joseph’s Health Care London - Parkwood Institute, 550 Wellington Rd, London, ON N6C 0A7 Canada; 7 Department of Family and Community Medicine, University of Toronto, 160‑500 University Avenue, Toronto, ON M5G 1V7 Canada; 8 Toronto General Hospital, University Health Network, 585 University Avenue, Toronto, ON M5G 2N2 Canada; 9 Institute of Health Policy, Management and Evaluation, University of Toronto, 155 College Street, 4th Floor, Toronto, ON M5T 3M6 Canada; 10 Central East Stroke Network, Royal Victoria Regional Health Centre, 201 Georgian Drive, Barrie, ON L4M6M2 Canada; 11 School of Physical Therapy, Western University, 1151 Richmond Street, London, ON N6A 3K7 Canada

**Keywords:** Community, Stroke, Randomized controlled trial, Task-oriented training, Healthcare-community partnership, Balance, Mobility, Everyday function

## Abstract

**Background:**

Despite the potential for community-based exercise programs supported through healthcare-community partnerships (CBEP-HCPs) to improve function post-stroke, insufficient trial evidence limits widespread program implementation and funding. We evaluated the feasibility and acceptability of a CBEP-HCP compared to a waitlist control group to improve everyday function among people post-stroke.

**Methods:**

We conducted a 3-site, pilot randomized trial with blinded follow-up evaluations at 3, 6, and 10 months. Community-dwelling adults able to walk 10 m were stratified by site and gait speed and randomized (1:1) to a CBEP-HCP or waitlist control group. The CBEP-HCP involved a 1-h, group exercise class, with repetitive and progressive practice of functional balance and mobility tasks, twice a week for 12 weeks. We offered the exercise program to the waitlist group at 10 months. We interviewed 13 participants and 9 caregivers post-intervention and triangulated quantitative and qualitative results. Study outcomes included feasibility of recruitment, interventions, retention, and data collection, and potential effect on everyday function.

**Results:**

Thirty-three people with stroke were randomized to the intervention (*n* = 16) or waitlist group (*n* = 17). We recruited 1–2 participants/month at each site. Participants preferred being recruited by a familiar healthcare professional. Participants described a 10- or 12-month wait in the control group as too long. The exercise program was implemented per protocol across sites. Five participants (31%) in the intervention group attended fewer than 50% of classes for health reasons. In the intervention and waitlist group, retention was 88% and 82%, respectively, and attendance at 10-month evaluations was 63% and 71%, respectively. Participants described inclement weather, availability of transportation, and long commutes as barriers to attending exercise classes and evaluations. Among participants in the CBEP-HCP who attended ≥ 50% of classes, quantitative and qualitative results suggested an immediate effect of the intervention on balance, balance self-efficacy, lower limb strength, everyday function, and overall health.

**Conclusion:**

The CBEP-HCP appears feasible and potentially beneficial. Findings will inform protocol revisions to optimize recruitment, and program and evaluation attendance in a future trial.

**Trial registration:**

ClinicalTrials.gov, NCT03122626. Registered April 21, 2017 — retrospectively registered.

## Key messages regarding feasibility

1. **What uncertainties existed regarding the feasibility?**
  - Will recruitment of participants, interventions, retention, and data collection be feasible in a multi-site study?
  - Will participants find it acceptable to wait 10 months in the control group before being offered the exercise program?
  - Will the exercise program confer some benefit?
2. **What are the key feasibility findings?**
  - Recruitment was challenging but could be boosted by enlisting a healthcare professional within the circle of care to recruit.
  - A wait time of 10 months in the waitlist control group was unacceptably long.
  - The CBEP-HCP appeared feasible and beneficial.
  - Retention was acceptable but attendance at exercise classes and evaluations was inconsistent, mainly due to challenges with health, weather, and transportation.
3. **What are the implications of the feasibility findings for the design of the main study?**
  - A future definitive effectiveness trial of the CBEP-HCP is supported by acceptable intervention fidelity, potential intervention benefit, and retention. Findings can inform protocol modifications to improve recruitment, acceptability of the waitlist control group, and program and evaluation attendance.

## Background

Stroke remains a leading cause of long-term disability worldwide [1]. In Canada alone, over 400,000 people are living with the effects of stroke, a number that is estimated to reach 726,000 by 2038 [2]. After a stroke, people often experience balance and walking limitations [3–6] which contribute to decreased function in everyday activities, including basic and instrumental activities of daily living (ADL), in the community [7]. Reduced function has broad consequences. It negatively affects participation in meaningful activities, and health-related quality of life (HRQL) while increasing the need for caregiver assistance [8]. Decreased instrumental ADL function is also a significant predictor of hospitalization, home healthcare and social services utilization, and institutionalization in older adults [9, 10].

Although regular exercise participation following the rehabilitation phase would help to maintain or improve function, people post-stroke face personal (e.g., insufficient knowledge of how to exercise, fear of an adverse event, embarrassment, and lack of motivation [11–13]) and environmental (e.g., inadequate building/equipment access, insufficient instructor training/expertise, program cost, and lack of transportation) [12, 14] barriers to engaging in mainstream fitness programs. To address these barriers, community-based exercise programs (CBEPs) tailored to the needs of people with stroke have been developed [15–20] and offered as wellness programs in community settings [21]. Programs commonly incorporate group, task-oriented training, which involves the practice of functional activities, such as sit-to-stand, standing weight-shifts, and walking, for several reasons. Task-oriented training is effective in increasing balance, walking, cognition, and HRQL post-stroke [15, 19, 22–24]. Task-oriented training is feasible to implement in recreation settings by non-regulated fitness personnel as the exercises involve practice of familiar activities with straightforward progressions, and minimal human and equipment resources and costs when exercises are organized in a circuit [24]. Exercising in groups helps foster self-efficacy to perform functional tasks, social interaction, and motivation to exercise [25]. Some organizations offer CBEPs at no or little cost and provide subsidies [26, 27]. Furthermore, partnerships with healthcare professionals can facilitate education and training on stroke-related impairments and functional limitations and appropiate exercises for participants for the community center staff [28, 29].

Despite the potential benefits of group, task-oriented CBEPs that incorporate a healthcare-community partnership, implementation is hindered by program cost, challenges training fitness instructors, staff turnover, and inadequate referral and transportation [21]. Evidence of effectiveness from a rigorously designed trial is needed to inform health and recreation policy and justify implementation of the program model to governmental, community, charitable, and healthcare organizations. This evidence, however, is scarce. Results from one randomized trial [15] conducted in the UK showed improvement in everyday function post-program that was maintained at one year. Findings from a pilot trial [20] suggest CBEPs can improve walking speed, but not balance, mobility, or HRQL. Influence on other important outcomes, such as injurious falls, and caregiver assistance, is unknown. There is a need to incorporate strategies, such as stratification on study site as well as physical function, a known strong confounder [30], monitoring of co-interventions, and adjustment for clustering and confounding in the analysis, to optimize methodological rigor. Use of qualitative methods, particularly a mixed methods approach that involves the collection and analysis of quantitative and qualitative data to address study aims [31], is recommended in feasibility trials to obtain an in-depth understanding of experiences and quantitative findings [32, 33].

### Together in Movement and Exercise program

Together in Movement and Exercise *(*TIME^TM^) is a 12-week, community-based exercise program incorporating a healthcare-community partnership (CBEP-HCP) licensed by the University Health Network (UHN) [18, 34]. In this partnership, a healthcare professional provides standardized training to fitness instructors to deliver a group, task-oriented exercise program targeting balance and mobility in community centers. A healthcare professional (typically a physical therapist), called the healthcare partner, visits the exercise program to observe and provide feedback to instructors. People with balance and mobility limitations must be able to walk at least 10 m with or without mobility devices and without assistance from another person to participate. Involvement of a healthcare professional to train instructors and visit the program appears to help safeguard the safety and health benefits of the program [29]. Participants and caregivers perceive that balance, core, and leg strength, and confidence improve following the program, which contributes to improved walking, ability to use stairs, transfers, ADL function, participation in social and leisure activities, and reduced caregiver assistance [18, 25]. Evidence of safety and feasibility of the training and exercise program has been reported [18]. To date, however, a randomized controlled trial of this program model has not been undertaken in Canada to provide evidence that would help to influence widespread implementation, funding, and policy.

Prior to launching a large-scale trial, a pilot randomized controlled trial is required to assess methodological feasibility and acceptability of the study methodology. Thus, study objectives were to:

1. evaluate the feasibility and acceptability of a two-group randomized controlled trial evaluating the effectiveness of the TIME^TM^ program compared to usual care in improving everyday function in ambulatory people discharged home post-stroke;
2. describe the potential effects of the TIME^TM^ program compared to usual care; and
3. identify an outcome measure of everyday function based on sensitivity to change.

## Methods

### Study design

A two parallel-group, mixed methods, pilot randomized controlled trial was conducted in three urban centers (Toronto, Pembroke, and London) in Ontario, Canada from March 2017 to April 2019. Research ethics approval was obtained at each hospital site and the University of Toronto. Our reporting follows the CONSORT guideline [35]. A qualitative descriptive approach [36] was used to complement analysis of quantitative data to evaluate the feasibility and acceptability of recruitment, retention, data collection, and interventions, and effects of the exercise program [31].

### Eligibility and recruitment

In each center, a hospital with designated stroke rehabilitation beds formed a partnership with a recreation center. Hospital managers invited a registered physical therapist with at least one year of experience treating people with stroke to fulfill the role of the healthcare partner. Healthcare partners were expected to observe fitness instructor training sessions, train volunteers (where available), observe five TIME^TM^ classes in a 24-class session and provide a 15-min debrief to fitness instructors after the observed class. Recreation centers that were located within 50 km of the hospital to facilitate healthcare partner visits, fully accessible, in close proximity to public transport, with an appropriately sized multi-purpose room, and recreation programming for all ages and abilities, were considered eligible. Recreation managers identified three fitness instructors meeting the following criteria to deliver the TIME^TM^ program: group fitness instructor certifications, including CanFitPro™ Fitness Instructor Specialist, YMCA-Fitness Leadership, Ontario Fitness Council (OFC), American Council on Exercise (ACE), or equivalent; excellent communication and leadership skills; empathy, enthusiasm and a genuine interest in working with people with disability; and two volunteers to assist with setup and takedown of equipment, and supervise exercises in the walking station. Research team members (authors NMS and JH) met with each hospital and recreation provider to ensure centers met the requirements for the study and to deliver the TIME™ exercise program.

We targeted ambulatory adults living in the community post-stroke. Inclusion criteria were (1) clinical diagnosis of stroke documented in the health record; (2) age ≥ 18 years; (3) living at home for at least 3 months post-hospitalization for stroke to allow sufficient time to transition to community living; (4) self-reported ability to walk ≥ 10 m with or without walking aids without assistance from another person; (5) ability to follow verbal instructions and speak and read English as judged by the recruiter; and (6) willingness to sign a liability waiver verifying medical clearance from a healthcare provider, and noting that TIME^TM^ was intended as a wellness program, not as rehabilitation or physical therapy. Exclusion criteria were (1) self-reported involvement in another exercise or rehabilitation program; (2) self-reported conditions or symptoms (e.g., unstable cardiovascular disease, significant joint pain) preventing exercise participation; (3) cognitive or behavioral deficits that would prevent cooperation within a group, as judged by the recruiter; (4) self-reported ability to walk ≥ 20 min without a seated rest; and (5) self-reported ability to manage environmental barriers (curbs, ramps, and stairs) with relative ease.

Caregivers of consenting participants were invited to participate in the study to complete caregiver-related study measures and participate in qualitative interviews about study experiences. Caregivers were considered eligible if they (1) helped the individual post-stroke to live at home and provided support and assistance with at least one basic and /or instrumental ADLs at least once a week [37]; and (2) were able to speak and read English. Paid personal support workers were excluded.

Table 1 outlines the recruitment approach at each site. Recruiters obtained verbal informed consent, and scheduled baseline evaluations except for the Toronto site where the coordinator performed these tasks. Individuals with stroke and caregivers provided written informed consent at the baseline evaluation.

**Table 1:** Recruitment approach at study sites

Site	Nature of recruitment	Recruiter	Recruitment strategies
Toronto	Prospective	In-patient physical therapist	• Distributed study brochures to inpatients and outpatients and their caregivers March–September 2017 and screened for eligibility at discharge
	Retrospective	Out-patient physical therapist	• Contacted discharged outpatients seen October 2016–January 2017 by phone to screen for eligibility and gauge interest to participate
Pembroke	Retrospective	Stroke team nurse practitioner	• Contacted discharged outpatients seen January 2016–January 2017 by phone, mailed brochures and consent forms to interested individuals
London	Retrospective	Research assistant	• Contacted people who were inpatients or outpatients January–June 2017 by phone and mail

### Outcomes

The primary study outcome was everyday function. Secondary outcomes included life-space mobility, ADL function, HRQL, caregiver assistance, caregiver emotional health, and injurious falls. Explanatory outcomes, meaning those expected to help explain changes in everyday function, included balance, balance self-efficacy, lower limb strength, walking speed, walking distance, cognition, and depression.

#### Primary outcome

Everyday function was assessed using the subjective index of physical and social outcome (SIPSO) [38] and the Nottingham extended activities of daily living (NEADL) [39]. The SIPSO is a 10-item self-report questionnaire comprising two 5-item subscales developed to capture physical (e.g., dressing, daily activities at home) and social (e.g., communication, satisfaction with friendships) integration post-stroke [38]. Participants score each activity from 0 to 4 where a higher score indicates a better level of integration. Subscale and total scores can range from 0 to 20 and 0 to 40, respectively. In community-dwelling people with stroke, intraclass correlation coefficient (ICC) values were 0.91 indicating excellent test reliability of subscale and total scores. Correlations of scores on the SIPSO with scores on the Barthel index, Frenchay activities index, and Wakefield depression inventory ranging from 0.73 to 0.80 support the construct validity of SIPSO [38].

The NEADL is a 22-item, self-report measure of IADL performance with 4 scales: mobility, kitchen, domestic, and leisure. Item-level scores range from 0 to 3 where 0 indicates “unable” and 3 indicates “on my own”. Total scores can range from 0 to 66. Spearman’s correlations of repeated measures using the subscales and total score ranging from 0.83 to 0.93 indicate a high level of test-retest reliability [40] and correlations of 0.88–0.90 with scores on the Barthel Index and Frenchay activities index provide evidence of construct validity in community-dwelling people with stroke [41].

#### Secondary outcomes

Life-space mobility and independence with basic ADL were evaluated using the lifespace assessment (LSA) [42], and the 10-item Barthel index (BI) [43], respectively. The lifespace assessment scale assesses how far and how often individuals have mobilized in their immediate and distant living environment within the past 4 weeks. HRQL was evaluated using the stroke impact scale (SIS) [44], and the euroqol-5D-5L (EQ-5D-5L) [45, 46]. The EQ-5D-5L captures dimensions of mobility, self-care, usual activities, pain/discomfort, and anxiety/depression, and converts to a single index value that can be used to calculate quality-adjusted life-years (QALYs). A visual analog scale (EQ-VAS) yields a rating of current health that can range from 0 to 100 points. Caregiver assistance was assessed using the 17-item, self-report caregiver assistance scale (CAS) [47, 48]. Caregiver emotional health was evaluated using the Research ANd Development-36 (RAND-36) emotional well-being and energy/fatigue score [49]. An injurious fall was defined as “an unexpected event in which the participant comes to rest on the ground, floor, or lower-level” [50] which results in an injury requiring medical care [51]. Participants were provided with monthly falls log calendars to document the occurrence of falls. Participants were contacted monthly to identify fall occurrence and determine if the fall led to injury requiring medical care [50]. At each evaluation, participants were asked to report on participation in co-interventions (e.g., physical therapy, alternate exercise classes) since the last evaluation.

#### Explanatory outcomes

Balance, balance self-efficacy, lower limb strength, comfortable walking speed, walking distance, cognition, and depression were evaluated using the Berg balance scale (BBS) [52], activities-specific balance confidence (ABC) scale [53], 30-second timed sit-to-stand (30-STS) test [54], 10-m walk test (10mWT) [55], 6-min walk test (6MWT) [56, 57], trail making test (TMT) [58], and geriatric depression scale-short version (GDS) [59], respectively.

To evaluate intervention fidelity, we documented implementation of TIME^TM^ program elements, including the license, training (instructors, healthcare partners, volunteers), class frequency/duration, exercise class components (warm-up, specific exercises, cool down, recommended equipment, participant-to-instructor ratio), and healthcare partner visits. Fitness instructors documented attendance and adverse events that occurred during exercise classes using a standardized form.

Feasibility of recruitment was evaluated by computing site-specific recruitment rates (number recruited/recruitment period in months) and the percentage of caregivers agreeing to participate. Feasibility of retention and data collection was summarized using percentages of individuals in each group withdrawing, completing evaluations, and providing monthly falls data. Intervention fidelity was reported as the number of sites implementing program elements, the number and percentage of classes delivered, the percentage of classes in which the prescribed class format (i.e., warm-up, recommended exercises, cooldown) was followed. Participant engagement was determined by the percentage of classes attended. The number of participants receiving co-interventions was noted.

### Data collection

We planned for trained evaluators, blinded to study hypotheses and group assignment, to complete evaluations at 0, 3, 6, and 12 months at the hospital site. After baseline, participants were given the choice of completing self-report measures at home with each follow-up evaluation to shorten evaluation sessions.

At baseline, we collected data on participant age, sex, education level, employment status, income level, presence of caregiver, side of stroke, time post-stroke, comorbidity (Charlson comorbidity index [60]), frailty (Canadian study of health and aging frailty scale [61]), type of mobility aids and orthoses used, as well as caregiver age, sex, role, employment status, and time spent caregiving. Measures of primary, secondary, and explanatory study outcomes, with the exception of injurious falls, were administered at each evaluation.

After the three-month evaluation, the research coordinator (KB) invited exercise and caregiver participants to separately participate in site- and intervention-specific focus groups or interviews by telephone that lasted approximately 45 to 60 min. The research coordinator conducted all interviews. KB is female and has a Master of Arts degree in Human Kinetics and six years of experience with conducting qualitative research. She explained the purpose of the interview, allowed participants to ask questions, and obtained verbal consent before proceeding. Participants were asked about their experiences with the study. Sessions were digitally recorded and professionally transcribed verbatim. The research coordinator reviewed transcripts for accuracy.

### Randomization

Participants were stratified by site, and level of comfortable gait speed deficit (severe: ≤ 0.5 m/s; mild-moderate: > 0.5 m/s) and block randomized to either the TIME^TM^ program (i.e., immediate group) or waitlist group in a 1-to-1 allocation ratio after the baseline evaluation. A Toronto-based research assistant, unfamiliar with participants, prepared a list of randomization assignments for each site by flipping a coin (block size of 2) and informed participants of group allocation by phone. We stratified by gait speed as it has been previously shown to modify the effect of task-oriented training on walking capacity post-stroke [30]. Blocking was used to balance the size of study groups to maximize statistical efficiency [62].

### Intervention

The program involves two 1-h exercise classes per week for 12 weeks. Each 1-h class involves a seated warm-up, repetitive and progressive practice of functionally relevant balance and mobility tasks, and a seated cool down. Warm-up consists of active range-of-motion exercises, aerobic exercise, lower extremity weight-bearing, stretching, and sit-to-stand training. The cooldown involves exercises similar to the warm-up but with an emphasis on stretching and relaxation. Participants, grouped by ability level, complete exercises organized in a 3-station circuit as follows: station 1: walking, aerobic training, and wall work (standing and reaching, wall push-ups); station 2: standing weight shifts, stepping, and lunging; and station 3: tap-ups, step-ups, and heel/toe raises, hamstring curls, marching-on-the-spot, and mini-squats. Each exercise has several levels of challenge to enable tailoring by instructors. Instructors are advised to have participants exercise at an intensity of 3–4 (moderate to somewhat hard) on the modified Borg scale [63]. Volunteers can be used to achieve the instructor plus volunteer-to-participant ratio of 1-to-4 to ensure adequate supervision and safety. The class is modeled after task-oriented interventions delivered by healthcare professionals with evidence of safety, feasibility, and efficacy from randomized trials involving people post-stroke [16, 30, 64–66]. Caregivers of participants with severe motor deficits are encouraged to assist as needed during the class.

After signing the license, the recreation provider receives an electronic toolkit with materials required to implement the program. Materials include the participant referral form and liability waiver, exercise guidelines, equipment/resource list, and measures to use for program evaluation. TIME^TM^ trainers delivered a 6-h in-person training workshop observed by the fitness instructors and healthcare partners that involved describing the program model, the roles of the partners, and movement challenges experienced by exercise class participants, and review and practice of the exercises including all levels of challenge. Volunteers were asked to view a video of the TIME^TM^ program, review the exercise guideline, and complete a 1.5-h session with the healthcare partner or a fitness instructor to review the program, their role and responsibilities, and practice the exercises in the walking station.

Healthcare partners were instructed to visit the first 2 classes to advise fitness instructors on participant grouping, safety considerations and exercise modifications, and three more classes spread out over the remaining 11 weeks of the program, and address any questions from the fitness instructors by email or phone. The waitlist group received usual care and were offered to participate in the 3-month exercise program following the final evaluation.

### Sample size

We proposed to recruit 20 participants (and their caregivers if available) per site for a total sample size of 60. The exercise class runs with a group of 8–12 participants. Thus, a sample size of 60 was considered sufficient to enable trialing of the exercise program in small and large urban centers with a similar exercise class size to that planned for the exercise program in the definitive cross-Canada trial. A sample size of 30 participants per group provided 80% power to detect an effect size of 0.72 for the SIPSO-Physical (given SD = 1 [15]) [67].

### Analysis

As this was a pilot study, we did not test hypotheses related to the effectiveness of the TIME^TM^ program compared to usual care. Participant data were analyzed in the group to which participants were randomized. We summarized scores on each outcome measure by group using medians and 25th and 75th percentiles for continuous data and with frequencies and percentages for categorical data at each evaluation time point. The risk of injurious falls during TIME^TM^ classes was estimated by computing the absolute risk difference (i.e., proportion of participants in the immediate group with an injurious fall minus proportion of participants in the waitlist group with an injurious fall). For a multi-item measure, if more than 10% of the items were missing data, the entire measure was considered missing. If ≤ 10% of items were missing, missing values were replaced by the average across other items in the scale and the total score was calculated. To identify the optimal measure of everyday function, we compared the effect size of ADL measures if data were normally distributed; otherwise, we examined change in scores from baseline to 3 months.

Transcripts were entered into NVivo to assist with the organization and analysis of the data. Using a directed content analysis [68], transcripts were coded based on the protocol element (i.e., recruitment, retention, data collection, and interventions), or the effect of the intervention, using a deductive approach. Authors KB and NMS independently reviewed and coded two transcripts and then met to discuss coding. KB then coded the remaining transcripts using NVivo10. Rigor was optimized by triangulating results from participants and caregivers and quotations were used to support identified themes. Results from quantitative and qualitative analyses for each objective were compared and contrasted to enhance the rigor and robustness of the analysis.

## Results

Across sites, 33 people with stroke (i.e., participants) and 13 caregivers consented to participate. Sixteen participants with 8 caregivers were randomized to the TIME^TM^ program, and 17 participants with 5 caregivers were randomized to the waitlist group. Table 2 presents baseline sociodemographic and clinical characteristics of participants and caregivers by intervention group.

**Table 2:** Baseline characteristics of participants and caregivers randomized vs included in the analysis by intervention group

Characteristic	Randomized (*n* = 33)		Analyzed (*n* = 21)	
	Immediate group (*n* = 16)	Waitlist group (*n* = 17)	Immediate group (*n* = 8)	Waitlist group (*n* = 13)
Age in years, median (P_25_, P_75_)	71 (65, 80)	67 (58, 79)	72 (66, 80)	65 (57,79)
No. (%) female	7 (43)	8 (47)	3 (38)	7 (54)
Level of education, No. (%)				
Secondary school or lower	9 (56)	12^b^ (70)	4 (50)	9 (75)
College	6 (38)	2 (12)	3 (27)	2 (17)
Graduate or Post-graduate	1 (6)	1 (6 )	1 (13)	1 (8)
No. (%) employed	1 (6)	1 (6)	1 (6)	1 (6)
Financial status				
Some money left over	10 (62)	3 (18)	7 (87)	2 (15)
Just enough to make ends meet	5 (32)	8 (47)	1 (12)	6 (46)
Not enough to make ends meet	0 (0)	3 (18)	0 (0)	3 (23)
Refused to answer	1 (6)	3 (18)	0 (0)	2 (15)
Side of stroke, No. (%)				
Right	5 (31)	9^a^ (53)	2 (25)	7 (54)
Left	10 (63)	7 (41)	5 (63)	6 (46)
Bilateral	1 (6)	0 (0)	1 (12)	
Months post-stroke, median (P_25_, P_75_)	12 (7, 17)	11 (7, 18)	13 (8, 26)	12 (8, 18)
No. (%) 6–12 months	8 (50)	10 (58)	4 (50)	6 (46)
No. (%) 12–18 months	4 (25)	4 (24)	2 (25)	4 (30)
No. (%) > 18 months	4 (25)	3 (18)	2 (25)	3 (23)
Charlson comorbidity index score, median (P_25_, P_75_)	4 (3, 6)	4 (3, 5)	4 (3, 5)	4 (3, 5)
Canadian study of health and aging frailty scale level, No. (%)				
Very fit	0 (0)	0 (0)	0 (0)	0 (0)
Well	1 (6)	1 (6)	1 (13)	1 (8)
Well with treated comorbid disease	3 (19)	1 (6)	1 (13)	1 (8)
Apparently vulnerable	3 (19)	6 (35)	1 (13)	5 (39)
Mildly frail	4 (25)	6 (35)	2 (25)	3 (23)
Moderately frail	4 (25)	3 (18)	2 (25)	3 (23)
Severely frail	1 (6)	0 (0)	1 (13)	0 (0)
Very severely frail	0 (0)	0 (0)	0 (0)	0 (0)
Terminally ill	0 (0)	0 (0)	0 (0)	0 (0)
Usual walking aids, No. (%)				
None	2 (13)	5 (29)	2 (25)	4 (31)
Single point cane	5 (31)	5 (29)	2 (25)	4 (31)
Quad cane	3 (19)	0 (0)	2 (25)	0 (0)
Hemi-walker	4 (25)	3 (18)	1 (13)	2 (15)
4-wheeled walker	2 (12)	4 (24)	1 (12)	3 (23)
No. (%) using AFOs	3 (38)	2 (14)	3 (38)	1 (7)
No. (%) with a caregiver	8 (50)	11 (65)	7 (87)	11 (84)
Caregiver participants				
No. (%) of caregivers recruited	8 (100)	5 (55)	6 (85)	5 (45)
Caregiver role				
Spouse/partner	6 (75)	4 (66)	5 (83)	4 (80)
Child	2 (25)	2 (33)	1 (16)	1 (20)
No. (%) female	2 (25)	3 (50)	2 (33)	3 (60)
Age in years, median (P_25_, P_75_)	68 (61, 74)	69 (63, 74)	69 (58, 74)	68 (58, 74)
No. (%) caregivers employed	1 (8)	0 (0)	1 (17)	0 (0)
Time spent caregiving, no. (%)				
0–9 h/week	2 (25)	2 (40)	2 (33)	2 (40)
10–20 h/week	1 (13)	0 (0)	0 (0)	0 (0)
> 20 h/week	5 (62)	3 (60)	4 (66)	3 (60)

*Abbreviations*: *P*_*25*_ 25th percentile, *P*_*75*_ 75th percentile, *No*. number, *AFO* ankle-foot orthosis

^a^Information missing for one participant

^b^Information missing for two participants

**Fig. 1:**
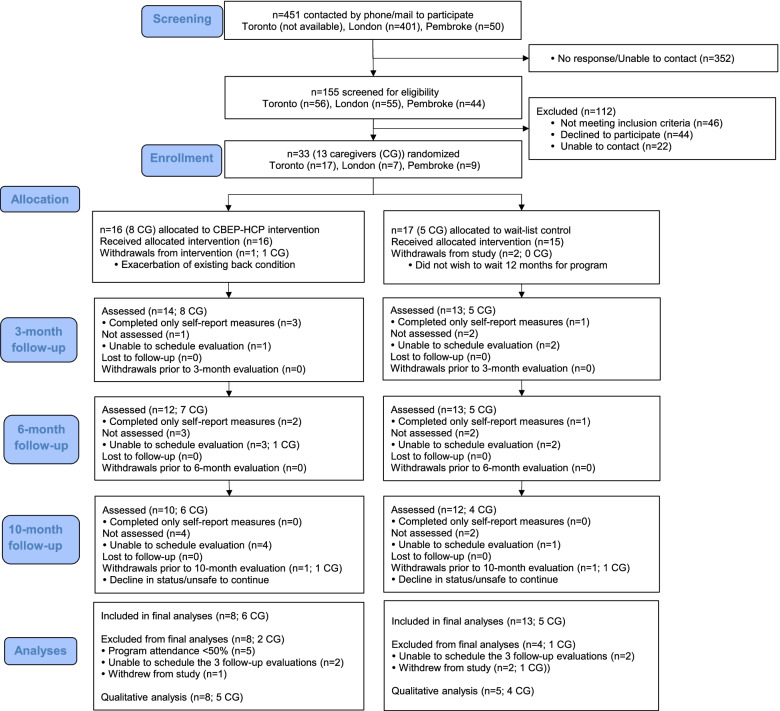
CONSORT flow diagram

Figure 1 describes the flow of participants through the trial. Participants remained in the group to which they were assigned. Thirteen participants (8 from immediate and 5 from waitlist group) and 9 caregivers (5 from immediate and 4 from waitlist group) from across sites participated in 16 interviews or focus groups. Participants interviewed included 8 men and 5 women, 55–83 years of age, who had attended either 3 (*n* = 2) or 4 (*n* = 11) evaluations. TIME^TM^ program participants interviewed had attended 72% of exercise classes. Caregivers interviewed included 5 men and 4 women, 44–72 years of age. One caregiver was a child and eight were spouses.

### Recruitment-quantitative

Across the 3 sites, 155 individuals with stroke were screened for eligibility. The period of recruitment was 7 months in Toronto and Pembroke and 6 months in London. Recruitment rates in Toronto, Pembroke, and London were 2.4, 1.2, and 1.1 participants/month, respectively. Of the 19 participants who had a caregiver, 13 (68%) agreed to participate.

### Recruitment-qualitative

A majority of participants reported having no preference for the method of initial contact (i.e., in-person vs. phone vs. mail) but indicated that having a member of their healthcare team recruiting facilitated their decision to enroll. Participants expressed the need for recruiters to describe the type, and benefits of the TIME^TM^ exercises to facilitate decision making. Caregivers indicated that recruitment materials did not clearly explain the caregiver’s role in the study. They recommended developing caregiver-specific recruitment materials that highlighted the social and physical health benefits of the program, and the opportunity for caregivers to participate in the exercise program to support their care recipient and/or “learn some of the moves” to practice them at home.

### Retention-quantitative

In the waitlist group, of the three withdrawals, two participants withdrew right after randomization as they were unwilling to wait 12 months to receive the intervention. For this reason, the research team decided mid-trial to reduce the wait period to 10 months. Prior to the 10-month evaluation, the research team withdrew a third participant (and their caregiver) who experienced significant decline in physical and cognitive ability, resulting in a final retention rate in the waitlist group of 82.4%. Two people in the TIME^TM^ program group withdrew. One participant (and their caregiver) withdrew after the first class due to an exacerbated back injury. The research team withdrew a participant (and their caregiver) prior to the 10-month evaluation who experienced a significant decline in physical and cognitive abilities, resulting in a final retention rate of 87.5%.

### Retention-qualitative

Retained participants and caregivers in the waitlist group reported that it was acceptable to be randomized to the waitlist group. The majority described wait times of 3 to 6 months as acceptable. Wait times of 10 or 12 months were considered “too long” as there would be no improvement if participants were “sitting around doing nothing”.

### Data collection and TIME^TM^ program implementation-quantitative

Table 3 provides an overview of participant and caregiver attendance at evaluations, completion of falls monitoring, and participant involvement in co-interventions. Table 4 describes characteristics of participating recreation and healthcare centers, program delivery staff, and TIME^TM^ program intervention fidelity. All recreation sites signed the TIME^TM^ license and completed training. Recreation staff and the healthcare partner from Toronto and Pembroke attended one-day training workshops. In London, fitness instructors, and a volunteer completed an e-learning module that reviewed knowledge-based content and also attended a 3-h training workshop. TIME^TM^ trainers oriented the London-based healthcare partner by Skype. Healthcare partners trained two volunteers in Toronto. The Pembroke site chose not to recruit volunteers. One instructor went on leave prior to delivery of the exercise program in the waitlist group at one site necessitating training of a new instructor.

**Table 3:** Completion of evaluations and falls monitoring and receipt of co-interventions

Protocol element	Immediate group (*n* = 16)	Waitlist group (*n* = 17)
Participant attendance at evaluations, No. (%)		
Baseline	16 (100%)	17 (100%)
3 months	14 (88%)	13 (76%)
6 months	12 (75%)	14 (81%)
10 months	10 (63%)	12 (71%)
Duration of falls data collection, No. (%)		
0-3 months	1 (6%)	3 (18%)
4-7 months	4 (25%)	1 (6%)
8-10 months	11 (69%)	13 (76%)
Receipt of co-interventions, No. (%)		
3 months	3 (19%)^**a**^	0 (0%)
6 months	2 (13%)^b^	4 (24%)^c^
10 months	0 (0%)	0 (0%)
Caregiver attendance at evaluations, No. (%)		
Baseline	8 (100%)	5 (100%)
3 months	8 (100%)	5 (100%)
6 months	7 (88%)	5 (100%)
10 months	6 (75%)	4 (80%)

^a^Physical therapy for second stroke (*n* = 1) or shoulder pain (*n* = 1); home exercise program for a severe foot drop (*n* = 1)

^b^Group pool exercise program at a recreation center (*n* = 2)

^c^Physical therapy to improve stroke recovery (*n* = 3); 3-month group exercise program (*n* = 1)

**Table 4:** Characteristics of recreation and healthcare centers, program delivery staff, and TIME^TM^ program implementation at three study sites

Characteristic	Study site		
	Toronto	London	Pembroke
Type of recreation organization	For-profit recreation center	Non-profit recreation center	For-profit fitness center
No. fitness instructors trained	3	4	3
No. volunteers trained (qualifications)	2 (undergraduate students)	1 (retired physical therapist)	0
Class schedule	Mon: 10:45–11:45 am Fri: 9:30–10:30 am	Tues & Thurs: 2:00–3:00 pm	Mon & Wed: 2:00–3:00 pm
Program dates	Oct 2017–Jan 2018	Oct 2017–Jan 2018	Apr–Jul 2018
Class size, Range	2–6	2–4	1–4
Instructor-plus-volunteer to participant ratio	1:4 or better	1:4 or better	1:4 or better
No. (%) of 24 classes delivered	22^a^ (92)	23^a^ (96)	24 (100)
Percent of classes adhering to prescribed class format	100	100	100
Transportation options utilized	Participant drove Caregiver drove Public adapted transportation Red Cross driving service^b^	Participant drove Caregiver drove Driving service^b^ (no public-adapted transportation available)	Participant drove Caregiver drove Driving service^b^ (no public-adapted transportation available)
Type of healthcare organization	Community general hospital with inpatient and outpatient stroke rehabilitation services	Regional stroke center with inpatient and outpatient stroke rehabilitation services	Regional stroke center with inpatient and outpatient stroke rehabilitation services
Role of decision-maker	Program director, medicine and post-acute services	Medical director, stroke rehabilitation program	Director, physical medicine and rehabilitation
Healthcare partner (mode of transportation to recreation site)	Physical therapist in outpatient program (car)	Physical therapist in outpatient program (car)	Physical therapist in outpatient program (car)
Distance to recreation center (km)	2.0 km	6.1 km	2.7 km
No. (%) of 5 healthcare partner visits completed	5 (100)	5 (100)	5 (100)

*Abbreviations*: *No.* number, *NA* not applicable

^a^Classes canceled due to public holidays

^b^Cost depended on distance traveled and ranged from $9 to $35 per trip

In the TIME^TM^ program group, 5 of the 16 participants attended fewer than 50% of the classes for health reasons that included a new diagnosis of stage 5 melanoma (*n* = 1); swollen feet and blisters (*n* = 1); recurrent stroke (*n* = 1); fall while attending a medical appointment leading to hip fracture and surgical repair (*n* = 1); and an inability to attend two classes a week due to extreme fatigue (*n* = 1). One participant withdrew from the study. For the other 10 participants, median attendance was 92% (range 72-100%). No injuries or incidents related to the intervention were reported.

### Data collection and TIME^TM^ program implementation-qualitative

Participants reported that attending four evaluations was acceptable. A phone call reminder a few days in advance of the evaluation was considered helpful. Some participants described the evaluations as lengthy and tiring, with repetition of some questions across questionnaires. Some preferred completing questionnaires online or over the phone prior to the evaluations, while others preferred completing them in-person so they could ask clarifying questions.

Across sites, participants and caregivers made positive comments about exercise instructors, describing them as empathetic, encouraging, and well-trained to teach and tailor the difficulty level of exercises to participant ability level, adapt exercises when necessary, and challenge participants in a safe manner. One participant commented:

They didn’t make us feel like we were stroke victims or anything. We were just normal people having an exercise program. They were very awesome about that. Like they didn’t discriminate on anybody’s level or anything. ~S2P-C1 (participant-waitlist control)

Some participants at the Toronto and London sites found visits from a familiar healthcare partner reassuring. One participant commented:

Oh, she was watching everything and making sure everything was going right. She encouraged people too…She knew most of us because I remember her from the [name] Clinic. She did her job…maybe suggested a few things to the instructors. But she was good. **~**S3P-E1 (participant-immediate group)

At all sites, some participants described challenges related to accessibility that decreased the acceptability of program delivery. Challenges included an inadequate number of accessible parking spots, entrances inaccessible due to construction, and the occasional malfunction of accessible doors. At one site, the two weekly classes were held in different rooms which caused some confusion. At another site, the class was run in a fitness studio where another fitness class was occasionally run concurrently which was distracting.

Participants across sites described challenges with transportation to the program and evaluations. Challenges includes long, tiring commutes (1.5–2 h one-way), transferring transit systems, and delays or no-shows of public transit services. Driving in snowy or icy weather conditions, and cost of hiring a driving service or a personal support worker to accompany them, further impacted participants’ abilities to attend the program. Nevertheless, participants and caregivers at all sites expressed a willingness to commute long distances to access the program due to its potential benefits, and the lack of similar programs in their communities. One participant commented:

Yeah, for me it’s an hour and a half from door to door… it's either go or don’t go…to be honest, the call was going to be sure, we’ll try it a couple of times. If it’s… a waste of time, you know, if we don’t feel it’s beneficial, we’re going to pull the plug. But it was worthwhile so we bit the bullet and drove 3 hours a day to go. ~S2P-E1 (participant-immediate group)

Recommendations included increasing the class duration to make a long commute worthwhile, offering more sessions per week, running the program during good weather months, and providing a home-based program. One caregiver described the challenges of attending the program during cold weather:

Like even taking him to the [recreation centre] in the wintertime, like it was freezing there. So…it would be better…to have [these programs] in the nice weather as opposed to like say December until March. They should start in April and go until like maybe the end of October. It’s easier for the people to get around…Because… some people are in walkers and wheelchairs…so in the wintertime, it’s a little hard for them. ~S1CG-E1 (caregiver-immediate group)

### Potential effect-quantitative

To evaluate potential immediate and sustained effects, we excluded from the analysis five participants in the immediate group who did not attend at least 50% of the classes due to serious health conditions; five participants with missing data as they withdrew from the study (2 from immediate group, and 3 from waitlist group); and two participants in the waitlist group who did not attend any follow-up evaluations. Data from 8 participants and 6 caregivers in the immediate group, and 13 participants and 5 caregivers in the waitlist group were analyzed. Table 2 presents baseline characteristics and Table 5 presents median scores on outcome measures at each evaluation by intervention group. Compared to participants in the waitlist group at baseline, participants in the immediate group were older and had lower median scores on measures of physical function including the BBS, 30-STS test, 10mWT, and 6WMT. Scores on primary outcome measures of everyday function were not normally distributed. Change from 0 to 3 months in median score on the SIPSO and NEADL in the immediate vs waitlist group were: SIPSO-Physical (3 vs 0 points); SIPSO-Social (− 2 vs − 1 points); and NEADL: (-3 vs 10 points). The gains in median score on secondary outcome measures from 0 to 3 months in the immediate vs waitlist group were largest for EQ-5D-5L health utility (0.2 vs − 0.1); EQ-VAS (20 vs 10 points); SIS recovery VAS (23 vs − 5 points); SIS hand function (7 vs 1 point); and Barthel index (15 vs 0 points). To help explain results related to everyday function, we identified that the median score on explanatory outcome measures improved in the immediate group from 0 to 3 months for the BBS (+ 5 points), ABC scale (+ 22 points), 30-STS test (+ 3 points), and TMT-B ( − 39 s), but not the 6MWT, 10mWT, or TMT-A. No injurious falls occurred during exercise classes. One participant in the waitlist group experienced an injurious fall in month four while performing usual activities and sought the advice of their physician.

**Table 5:** Scores on primary, secondary, and explanatory outcome measures by intervention group across evaluation time points

Measure (score range)	Immediate Group (8 participants, 6 caregivers) Scores are median (P_25_, P_75_)								Waitlist Group (13 participants, 5 caregivers) Scores are median (P_25_, P_75_)							
	Baseline		3 months		6 months		10 months		Baseline		3 months		6 months		10 months	
	*n*	Score	*n*	Score	*n*	Score	*n*	Score	*n*	Score	*n*	Score	*n*	Score	*n*	Score
SIPSO Physical (0–20)	7	5 (3, 15)	8	8 (3, 16)*	8	7 (3, 16)*	7	**7 (5, 16)**	13	11 (9, 16)	13	11 (10, 16)	13	11 (5, 16)	10	**14 (9, 16)**
SIPSO Social (0–20)	8	14 (11, 15)	8	12 (10, 14)	8	14 (10, 16)	7	14 (8, 14)	13	14 (9, 17)	13	13 (9, 16)	13	12 (8, 14)	10	12 (9, 14)
NEADL (0–66)	8	31 (15, 52)	7	28 (12, 51)	6	30 (17, 53)	7	**32 (16, 49)**	13	39 (29, 53)	10	**49 (25, 53)**	10	**43 (23, 53)**	11	**41 (35, 50)**
LSA (0–120)	8	44 (27, 62)	8	41 (23, 72)	7	33 (29, 78)	7	34 (12, 54)	12	39 (21, 61)	13	29 (17, 52)	12	39 (22, 45)	10	**40 (27, 69)**
Barthel index (0–100)	7	80 (50, 100)	7	**95 (75, 100)**	8	**90 (51, 98)**	7	**95 (75, 100)**	12	95 (81, 99)	13	95 (83, 100)	13	95 (71, 100)	11	95 (85, 100)
SIS Physical (4–20)	8	10 (8, 15)	8	10 (9, 15)	8	10 (8, 16)	7	**11 (8, 16)**	13	12 (10, 15)	13	**14 (11, 16)**	12	12 (9, 14)	10	**13 (9, 16)**
SIS Memory (7–35)	8	32 (26, 34)	7	32 (27, 35)	8	31 (27, 35)	7	**33 (26, 35)**	13	29 (26, 35)	13	29 (27, 34)	13	29 (23, 32)	10	**30 (30, 31)**
SIS Mood (9–45)	8	33 (32, 34)	8	33 (28, 37)	8	32 (3, 35)	7	31 (30, 33)	13	33 (30, 35)	13	31 (29, 33)	13	33 (30, 36)	10	33 (28, 34)
SIS Communication (7–35)	8	30 (26, 35)	7	**32 (26, 35)**	8	**33 (29, 35)**	7	**32 (26, 35)**	12	30 (25, 35)	13	**32 (26, 35)**	13	**31 (25, 34)**	10	**33 (29, 34)**
SIS ADL (10–50)	7	31 (26, 45)	6	**34 (23, 35)**	8	**33 (22, 46)**	7	31 (24, 45)	13	39 (31, 44)	12	**42 (35, 46)**	13	39 (24, 48)	10	**42 (38, 44)**
SIS Mobility (9–45)	8	32 (24, 40)	8	**34 (26, 41)**	8	30 (26, 37)	7	32 (29, 34)	13	35 (28, 39)	12	35 (26, 41)	12	31 (28, 39)	10	**39 (28, 42)**
SIS Hand Function (5–25)	8	10 (5, 23)	8	**17 (8, 23)**	8	**13 (6, 20)**	7	**11 (6, 24)**	13	17 (11, 20)	12	**18 (13, 22)**	13	**18 (13, 21)**	10	**18 (14, 22)**
SIS Participation (8–40)	7	21 (17, 35)	8	**32 (18, 34)**	8	**23 (15, 33)**	7	**35 (18, 37)**	13	28 (24, 34)	12	**34 (25, 38)**	13	**30 (28, 36)**	10	27 (20, 33)
SIS Recovery VAS (0-100)	6	40 (28, 90)	8	**63 (46, 90)**	8	**50 (20, 94)**	7	**60 (50, 90)**	9	75 (53, 85)	9	70 (53, 78)	13	70 (53, 85)	10	**80 (58, 90)**
EQ-5D-5L Health Utility	7	0.5 (0.4, 0.7)	8	**0.7 (0.6, 0.8)**	8	**0.6 (0.5, 0.7)**	7	**0.6 (0.5, 0.8)**	13	0.8 (0.6, 0.9)	13	0.7 (0.6, 0.8)	13	0.8 (0.5, 0.8)	10	0.8 (0.5, 0.8)
EQ-5D-5L VAS (0–100)	7	60 (50, 75)	8	**80 (70, 94)**	8	**78 (40, 84)**	7	**75 (50, 85)**	12	70 (62, 75)	12	**80 (67, 84)**	13	**80 (50, 80)**	10	**80 (50, 83)**
CAS (0-102)	6	52 (31, 84)	6	53 (35, 78)	6	52 (34, 83)	5	**33 (16, 55)**	5	54 (32, 71)	5	78 (33, 84)	5	57 (25, 75)	4	60 (38, 82)
RAND Emotion (0–100)	6	62 (57, 76)	6	**64 (55, 64)**	6	**70 (61, 76)**	5	**74 (49, 84)**	4	62 (59, 68)	5	**76 (64, 84)**	5	56 (52, 76)	4	**64 (40, 64)**
RAND Fatigue (0–100)	6	43 (25, 49)	6	33 (26, 39)	6	35 (47, 43)	5	**53 (41, 68)**	4	50 (44, 55)	5	35 (30, 40)	5	50 (30, 55)	4	50 (30, 75)
Berg Balance Scale (0-56)	8	35 (22, 48)	7	**40 (33, 50)**	7	**49 (26, 52)**	7	**45 (26, 51)**	13	44 (27, 49)	13	**47 (30, 52)**	11	**46 (23, 52)**	11	**48 (38, 52)**
ABC Scale (0–100)	7	39 (28, 76)	8	**61 (32, 79)**	8	**49 (32, 71)**	7	**53 (34, 83)**	12	65 (57, 82)	12	**72 (60, 91)**	13	63 (34, 77)	10	**69 (53, 83)**
30-sec Sit to Stand	8	3 (0, 8)	7	**6 (2, 9)**	7	**5 (0, 7)**	6	**6 (0, 9)**	12	6 (2, 8)	13	6 (0, 10)	11	5 (4, 9)	10	**7 (3, 9)**
10-m walk test (m/s)	8	0.5 (0.1, 0.9)	8	0.5 (0.1, 1.2)	7	0.5 (0.1, 1.1)	7	0.3 (0.1, 0.9)	13	0.7 (0.5, 0.9)	13	**0.8 (0.5, 1.1)**	11	0.7 (0.3, 0.8)	11	**0.8 (0.6, 1.1)**
6-min walk test (m)	8	157 (37, 320)	8	130 (102, 300)	7	**201 (82, 300)**	6	120 (95, 300)	13	241 (191, 299)	13	180 (112, 300)	11	174 (132, 300)	10	179 (114, 364)
Trail making test A (sec)	8	56 (45, 188)	8	56 (46, 275)	7	**47 (42, 95)**	7	**49 (44, 120)**	13	65 (38, 90)	11	**61 (43, 121)**	12	**51 (40, 170)**	11	**54 (36, 96)**
Trail making test B (sec)	8	169 (85, 300)	8	**130 (102, 300)**	7	**167 (62, 298)**	7	**120 (95, 300)**	13	202 (101, 300)	11	**180 (112, 300)**	12	**158 (130, 300)**	11	**142 (92, 300)**
GDS (0–15) No. (%)	8		8		8		7		12		12		13		10	
Normal (0–4)		0 (0)		**1 (13)**		0 (0)		**1 (13)**		0 (0)		**2 (15)**		**1 (8)**		0 (0)
Mild (5–8)		6 (75)		6 (75)		5 (63)		3 (38)		6 (54)		6 (46)		9 (69)		7 (70)
Moderate (9–11)		2 (25)		1 (13)		2 (25)		2 (25)		2 (15)		4 (31)		3 (23)		3 (30)
Severe (12–15)		0 (0)		0 (0)		1 (13)		1 (13)		4 (31)		0 (0)		0 (0)		0 (0)

*Abbreviations*: *SIPSO* subjective index of physical and social outcome, *NEADL* Nottingham extended activities of daily living, *LSA* lifespace assessment, *SIS* stroke impact scale, *ADL* activities of daily living, *VAS* visual analog scale, *EQ-5D-5L* EuroQol-5D, five-level, *CAS* caregiver assessment scale, *ABC* activities-specific balance confidence, *GDS* geriatric depression scale. Within each timeline column, the first sub-collumn represents the number of participants for which data were available

Bolded values reflect median scores at 3, 6, and 10 months that were improved compared to baseline

### Potential effect-qualitative

A majority of participants and caregivers in the immediate group described a range of benefits of the TIME^TM^ program. These included providing participants with a meaningful activity and an opportunity to socialize. One caregiver commented:

He’s in the house with me 24/7…. He doesn’t get to see anyone else all day long. … when I joined the study, I thought, okay, this is his opportunity to get out and have some interaction with other people. ~ S1CG-E1 (caregiver-immediate group)

Access to a formal program motivated participants to exercise, and reinforced caregivers’ efforts to engage the participant in physical activity. A majority of participants described improvements in their mobility, such as walking longer distances, at faster speeds, and with better form. Participants noted greater confidence and independence while walking while caregivers observed improvements in participants’ balance, transitions to using a less supportive walking aid (e.g., walker to cane), with benefits extending to daily activities, such as cooking and dressing. One participant noted:

And my balance was improved that I could do more things around the house. Like stand at the kitchen counter and cut up the onions and things for dinner, and start doing more of that. ~S2P-E1 (participant-immediate group)

Caregivers did not describe any reduction in the level of assistance they provided the care recipient as a result of program participation. They appreciated the opportunity to exercise alongside their family member to “be involved” and provide motivation to be more active. One caregiver commented:

It’s a win-win situation. You know, it builds up his physical and mental…strengths that he has it in him…And for me, I see that it’s good for him. Like I’m seeing that he’s actually showing an honest interest [in exercising]. ~S2CG-C1 (caregiver-waitlist control)

Caregivers who waited together outside the exercise class reported bonding over sharing similar experiences. One participant who primarily used a wheelchair and their caregiver reported not experiencing any change as a result of their participation in the program, possibly due to the participant’s low baseline status. Table 6 summarizes the challenges that were observed during the pilot study and the proposed changes to the protocol of the definitive RCT based on interpretation of quantitative and qualitative findings.

**Table 6:** Proposed revisions to the study protocol based on pilot results

Challenges during pilot study	Proposed changes to study protocol
*Recruitment* • Unable to achieve recruitment target of 20 per site.	• Engage a member of the stroke team preferably in the out-patient department, known to patients, to refer people to the study. • For prospective recruitment, ensure access to patients near the time of discharge. • Highlight the type and benefits of exercises in the program in recruitment materials. • Consider targeting other clinical populations to boost recruitment given the exercise program is not specific to any health condition.
• Some participants could not fully engage with the exercise program due to a low level of physical function, comorbidity, and cognitive decline.	• Revise eligibility criteria to require individuals to have the capacity to perform sit-to-stand independently, walk 10 m independently with or without a walking aid but without assistance or supervision of another individual, and pass a cognitive screen.
• Only 68% of caregivers were recruited.	• Develop caregiver-specific recruitment materials that highlight the role of caregivers in the exercise program and potential benefits for the caregiver.
*Length of wait time for control group*	
• 12-month waitlist period was too long and led to drop-outs and potentially co-interventions.	• Reduce the wait time in the control group to 6 months.
*Evaluations*	
• Inclement weather and inadequate access to transportation were perceived as barriers to attending the exercise program and evaluations. • Evaluations were considered lengthy. • Monthly follow-up calls for falls monitoring were challenging to complete for ~ 25% of participants.	• Schedule evaluations and intervention periods during good-weather months if possible. • Provide participants with information about transportation services available in their region at the time of recruitment. • Budget for reimbursement for parking, adapted transportation, and driving services for remote areas. • Provide participants with gift cards as an incentive to attend evaluations, and the option to receive an evaluation summary. • Streamline the number of study measures to reduce evaluation length. • Provide flexible data collection options for those unable to attend in person, e.g., administer self-report measures by telephone. • Remove monthly falls monitoring given the exercise program was deemed safe.
*Fitness instructor training*	
• Issues with fitness instructor availability necessitated identification and training of new instructors.	• Train 3–5 instructors annually per site to improve instructor availability and mitigate potential turnover.
*Program delivery*	
• Participants found it distracting when other classes were being run in the same room and when rooms and class times changed between sessions.	• Ensure no other classes are being run in the same room. • Recommend using the same room and time for both classes each week.
*Potential effect*	
• Improvement on measures of walking capacity over the 3-month exercise program was not observed. • In new sites, fitness instructors and volunteers deliver the TIME^TM^ program for the first time during the experimental phase and may lack the expertise to progress participants.	• Incorporate additional practice of exercises for fitness instructors in the training workshop. • Have fitness instructors deliver the exercise program to an initial group of participants prior to randomization.

## Discussion

This study is the first to explore the feasibility and acceptability of a 2-group, RCT protocol designed to evaluate the TIME^TM^ program in people post-stroke in Canada. We identified protocol challenges that included a lengthy wait time in the waitlist group, and recruitment, evaluation, and retention of individuals with stroke. Participants and caregivers largely perceived the TIME^TM^ program as beneficial in improving balance, strength, mobility, and everyday functioning. We identified the physical component of the SIPSO as the optimal outcome measure of everyday function.

### Feasibility and acceptability of study protocol

Rates of participant and caregiver recruitment were low across sites. Recruitment led by a healthcare provider (in-person or over the telephone) within the circle of care was a more successful recruitment strategy compared to contact by a research assistant. Adding community-based strategies, such as posting advertisements in hospitals and churches, and including advertisements in seniors center newsletters, to complement hospital-based strategies, may help bolster recruitment [20].

A 12-month waitlist control design, initially proposed to track study outcomes in the long-term, led to dropouts and was perceived as too long by participants and caregivers. In contrast, an RCT of a similar exercise program model with a 12-month waitlist group conducted in the UK involving 243 people post-stroke [15] completed data collection in 84% of participants at 6 months, and 71% at one year. This superior 12-month retention compared to the current study may have resulted from use of convenient home-based assessments, a smaller test battery, and limiting the 12-month evaluation to mailing of self-report questionnaires. In a recent pilot RCT of a community-based, task-oriented exercise program run three times a week in the United States [20], only 58% of participants completed the 6-month intervention and evaluations compared to 70% of control group participants who received a 6-month seated exercise program. As in the current study, reasons for withdrawals included medical illness and transportation [20]. Reasons for even lower retention at one site included difficulty parking, lack of transportation, longer travel times, and lower socioeconomic status compared to other sites [20]. These factors highlight the implementation challenges of conducting a pragmatic trial of a CBEP in people with stroke who commonly experience additional health conditions [69]. For the definitive RCT, we propose a 6-month waitlist control, considered acceptable by many participants, and only three evaluations at 0, 3, and 6 months to minimize participant withdrawals. A 6-month waitlist may also help minimize participation in co-interventions which occurred to a similar degree in each group. To decrease evaluation burden, a parsimonious set of study measures will be achieved by eliminating duplicative measures of everyday function and the lengthy SIS. Providing transportation allowances, arranging for driving services or alternative modes of transportation for participants who are unable to drive, and scheduling the program outside of months with inclement winter weather may further minimize absenteeism and missing data.

Overall, the delivery of the CBEP-HCP in its intended format was feasible. All sites were able to organize the resources required to deliver the program. Instructor training was considered effective given participants and caregivers found the program safe, acceptable, and beneficial. Program challenges with room accessibility and availability can be addressed by scheduling classes at a consistent time and location.

### Program impact

In the current study, we emphasized the magnitude of within-group change given the between-group differences in sociodemographic characteristics, physical capacity, and health at baseline among participants included in the treatment-received analysis. Participants in the immediate group appeared to demonstrate meaningful changes in measures of hand function, lower limb strength, balance, mobility, and perception of extent of recovery at the end of the 3-month program compared to baseline status. While the greatest gains were seen at 3-month evaluations for most outcomes, participants in the immediate group continued to show improvements in leg strength, balance confidence, perception of extent of recovery at the 10-month evaluations.

Contrary to expectation, the immediate group did not improve in walking speed or endurance over time, despite self-reported improvements in balance confidence and reduced use of walking aids. In an RCT [20] of a similar group, task-oriented exercise CBEP run by exercise instructors three times a week for 6 months, a significant increase in 6MWT-based walking distance and speed, but not scores on the BBS, short performance physical battery [70], or SIS, was observed. When exercise classes have been provided only twice a week for 8 weeks, a significant increase in timed “up and go” scores was not observed [15]. In the current study, fitness instructors at all sites and volunteers at one site were running the TIME^TM^ exercise program for the first time and may not have sufficiently challenged participants. A strategy used in one RCT [20] to mitigate a lack of experience among novice exercise instructors involved providing an opportunity to instruct an initial group of participants prior to beginning randomization. This strategy, combined with incorporating more time to practice exercises during training, may help promote appropriate tailoring of exercise difficulty level to participant ability.

An important design element of the program was the social interaction it afforded individuals with stroke facing similar challenges. Participants in the immediate group, however, did not demonstrate improvement in social functioning on the SIPSO-Social scale. One explanation is that all participants were recruited from tertiary hospitals which serve large geographical areas and transportation was time-consuming for many. In other TIME^TM^ programs, recreation staff create opportunities for interactions (such as post-program social time, end-of-program potlucks [18]) which may help to create a sense of community with the other participants.

Caregivers in the immediate group, who described improvements in participants’ balance, strength, and functioning, did not note reductions in caregiver assistance or improvement in emotion/fatigue until the 10-month evaluation. It is possible that improvements were limited to performance of basic but not instrumental ADL which necessitated the continued assistance of the caregivers. Additionally, accompanying and driving participants to the TIME^TM^ program twice a week might have offset any reductions in perceived caregiver burden resulting from improved ADL function.

Compared to the NEADL, participants in the immediate group showed greater median change on the SIPSO-Physical. The SIPSO [38], which is specific to stroke, measures physical and social reintegration after stroke and captures the level of difficulty faced when performing physical and social activities. Alternatively, the NEADL is intended to be used as a record of activity performed in the last few weeks with or without help [71]. The NEADL is vulnerable to influences of inclement weather and pandemic-related restrictions to maintain social distancing. Some activities included on the NEADL, such as writing a letter, may be viewed as outdated. Finally, the SIPSO has a 5-point response scale which may capture smaller gains compared to the 4-point response scale of the NEADL. For these reasons, we identified the SIPSO as the optimal measure of everyday function for a definitive trial.

### Limitations of the study

Due to the small sample size obtained and missing data, only descriptive statistics could be computed to compare the effect of the program vs usual care on functional abilities of persons with stroke living in the community. Participants were not blinded to their group allocation which could have resulted in a bias, especially on self-report measures. The generalizability of the findings is limited by the use of strict eligibility criteria, as well as by issues related to seasonal program delivery (e.g., inclement weather) which affected attendance at the program and evaluations.

## Conclusions

The CBEP-HCP appears safe, feasible, and potentially beneficial for post-stroke individuals with balance and mobility limitations living in the community. Findings will inform protocol revisions to optimize recruitment, acceptability of the waitlist control group, and program and evaluation attendance in a future trial.

## Data Availability

The datasets generated and/or analyzed during the current study are not publicly available due to small numbers and possible identification of individuals, but are available from the corresponding author on reasonable request.

## Abbreviations

10mWT: 10-m walk test
6MWT: 6-min walk test
ADL: Activities of daily living
ABC: Activities-specific balance confidence
BBS: Berg balance scale
BI: Barthel index
CCI: Charlson comorbidity index
CBEP-HCP: Community-based exercise program incorporating a healthcare-community partnership
GDS: Geriatric depression scale
HRQL: Health-related quality of life
IADL: Instrumental activities of daily living
ICC: Intraclass correlation coefficient
LSA: Lifespace assessment
NEADL: Nottingham extended activities of daily living
QALY: Quality-adjusted life years
RCT: Randomized controlled trial
SIPSO: Subjective index of physical and social outcome
SIS: Stroke impact scale
TIME^TM^: Together in Movement and Exercise
TMT: Trail making test
VAS: Visual analog scale
